# Nonequilibrium Green’s Functions for Functional Connectivity in the Brain

**DOI:** 10.1103/PhysRevLett.126.118102

**Published:** 2021-03-19

**Authors:** Francesco Randi, Andrew M. Leifer

**Affiliations:** 1 Department of Physics, Princeton University, Jadwin Hall, Princeton, New Jersey 08544, USA; 2 Princeton Neuroscience Institute, Princeton University, New Jersey 08544, USA

## Abstract

A theoretical framework describing the set of interactions between neurons in the brain, or functional connectivity, should include dynamical functions representing the propagation of signal from one neuron to another. Green’s functions and response functions are natural candidates for this but, while they are conceptually very useful, they are usually defined only for linear time-translationally invariant systems. The brain, instead, behaves nonlinearly and in a time-dependent way. Here, we use nonequilibrium Green’s functions to describe the time-dependent functional connectivity of a continuous-variable network of neurons. We show how the connectivity is related to the measurable response functions, and provide two illustrative examples via numerical calculations, inspired from *Caenorhabditis elegans*.

Understanding how neurons interact is fundamental to describing how their collective activity generates the dynamics of the brain. Advances in optogenetics and neuroimaging now allow activity to be stimulated in one neuron while simultaneously measuring the response of many others [1–3]. Functional connectivity encompasses the collection of strengths, signs, and time-varying properties that govern how a change in activity of one neuron affects another. Measuring functional connectivity at single-neuron resolution would constrain simulations by linking anatomical connectivity to neural dynamics. Further, measuring changes to functional connectivity can reveal how the brain changes with learning.

Most models of continuous-variable neural activity, like graded potentials or spiking rates, are formulated as differential equations [4–9]. They include parameters for local properties of direct connections in the network, such as synaptic strengths between two neurons. But those local properties cannot be measured directly in the network. Instead, experiments see an effective interaction between the two neurons, which includes contributions from indirect paths as well as the direct path.

An integral formulation, see, e.g., Ref. [10], is more convenient for transitioning between local direct connections and the effective ones that are more accessible experimentally. In the linear and time-translationally invariant (TTI) case (a condition we relax later), the activity *Ψ_i_* of neuron *i* is

(1) $$\Psi_{i}(t)=\Psi_{i,\text{eq}}+\underset{j\in \text{all}}{\sum }g_{0,ij}*\text{Δ}\Psi_{j}(t)+g_{0,i}^{\text{ext}}*I_{i}^{\text{ext}}(t),$$

where *Ψ*_eq_ are the equilibrium activities of the neurons and Δ*Ψ* the deviations from those values. * denotes convolution, *g*_0,*ij*_ is the (TTI) Green’s function, or transfer function, describing the direct interaction *i* ← *j* from neuron *j* to neuron *i*. $g_{0,i}^{\text{ext}}*I_{i}^{\text{ext}}\left(t\right)$ denotes the effect of external perturbations.

Equation (1) considers only direct paths between neurons. However, in a network *i* and *j* are connected by both direct and indirect paths, and one would have to solve Eq. (1) for each neuron and time step. If we know Δ*Ψ_j_* [11] and want to calculate Δ*Ψ_i_*, in a linear system we can condense the effect of the whole network into a single connected Green’s function $G_{0,ij}^{j}$ (the resolvent kernel in Volterra integral equations [12]), such that $\Psi_{i}\left(t\right)=\Psi_{i,\text{eq}}+\left(G_{0,ij}^{j}*\Delta \Psi_{j}\right)\left(t\right)$. $G_{0,ij}^{j}$ is a solution to

(2) $$G_{0,ij}^{j}=g_{0,ij}+\underset{\mu \neq j}{\sum }g_{0,i\mu }*G_{0,\mu j}^{j},$$

which is obtained by recursively inserting contributions of all neurons in Eq. (1). Upper case denotes connected, subscript 0 denotes linear and TTI, superscript *j* means *j* is excluded from summations. See Supplemental Material [13] for discussion of when Δ*Ψ_j_* is sufficient. Green’s functions govern the dynamics of the state ***Ψ*** = ***Ψ***_**eq**_ + **Δ*Ψ*** in the integral formulation of the system’s equations. To probe the system’s properties, we can induce a small perturbation *δΨ_j_* on top of the current state of the system ***Ψ*** and obtain the connected response function *F*_0,*ij*_ by measuring the produced *δΨ_i_* = *F*_0,*ij*_ * *δΨ_j_*. In the linear and TTI case, $F_{0,ij}=G_{0,ij}^{j}$, while they differ in a nonlinear system.

The brain is highly nonlinear. Nonlinearities allow it to perform nontrivial computations and incorporate past history or sensory context in its response. Green’s functions, however, are usually defined only for linear TTI systems, which may explain their limited use in neuroscience [4,10]. Nonlinear corrections to a Green’s functionlike formulation via systematic expansion has previously been used to describe the effect of hidden neurons [10] and spike train statistics [14] in spiking neurons. Because response functions are intuitive, and an experiment can always be designed to measure a response function, it is worth working with expanded, or corrected, Green’s functions.

We use nonequilibrium Green’s functions (NEGFs) *G_ij_* [14–17] to describe the time-dependent functional connectivity of a continuous-variable network of neurons, and discuss their relation to nonequilibrium response functions *F_ij_* measured in experiments (absence of subscript 0 means nonequilibrium). While they retain the benefits of transfer functions, their nonequilibrium definition as a function of relative *and* absolute time makes them well suited to capture nonlinearities and time dependence in the brain, for example, when synapses saturate, when synaptic adaptation occurs, or when neuromodulators alter neural properties over time. NEGFs appear in fields such as many-body theory in condensed matter physics, where they guide theory and experiment [15]. Here equilibrium refers to the time invariance of the Green’s functions, not the neural activities.

We first present a general model-independent equation for the connected nonequilibrium response functions *F_ij_* [Eq. (5)], that allow us to write $\delta \Psi_{i}\left(t\right)=F_{ij}*\delta \Psi_{j}=\int dt_{1}F_{ij}\left(t,t_{1}\right)\delta \Psi_{j}\left(t_{1}\right)$, and are obtained assuming sparse nonlinear connections, or edges (*α*, *β*). These edges are described with NEGF *g_αβ_*[***Ψ***] so that, formally, $\Delta \Psi_{\alpha }=g_{\alpha \beta }\left[\Psi \right]*\Delta \Psi_{\beta }=\int dt_{1}g_{\alpha \beta }\left[\Psi \right]\left(t,t_{1}\right)\Delta \Psi_{j}\left(t_{1}\right)$ for an isolated pair of neurons. Because *g_αβ_*[***Ψ***] is functionally dependent on the state ***Ψ*** of the system, it has to be calculated according to its nonlinear expression. Once calculated, however, other properties of the network, like the other Green’s functions and response functions, are easily derived and computed.

We describe how the *F_ij_*’s relate functional connectivity to experiments, apply this formalism to the nematode *Caenorhabditis elegans*, and illustrate general theoretical results with numerical calculations.

## Nonequilibrium response functions.—

As we derive an equation for the nonequilibrium response function *F_ij_*, we also address a seemingly puzzling observation about the *C. elegans* brain. Characterizations of some synapses in the worm show that they are linear throughout a large range of physiological activity [18–20]. However, we know that nonlinearities and time dependence are critically important in *C. elegans* and in nervous systems generally, because they allow the network to perform computations that cannot be captured by a linear system, including, e.g., responding to sensory stimuli in a context-dependent manner [21,22]. How does a network have many linear edges but also show widespread nonlinear behaviors? The NEGF integral formulation makes it straightforward to show how these two observations can coexist.

We start by considering a network in which only one edge (*α*, *β*) displays a significant nonlinearity. This is in contrast to the cases studied in Ref. [14], which have nonlinearities that are homogeneous over the network and that are systematically expanded. We will show how a time-dependent change of a single edge, due to a nonlinearity, can change effective connections and response functions elsewhere in the network.

The direct Green’s function $g_{\alpha \beta }\left(t,t^{\prime }\right)=g_{0,\alpha \beta }\left(t-t^{\prime }\right)+\pi_{\alpha \beta }\left[\Psi \right]\left(t,t^{\prime }\right)$ for the nonlinear or time-dependent edge can be written as the sum of a linear TTI term *g*_0,*αβ*_ and a nonequilibrium term *π*[***Ψ***], dependent on the system’s state ***Ψ***. For the isolated pair *α* ← *β*, *g_αβ_* allows one to calculate the response function *f_αβ_* that determines, in an effective linear regime, the *δΨ_α_* measured in an experiment after a perturbation *δΨ_β_* on top of the current state ***Ψ*** = ***Ψ***_**eq**_ + **Δ*Ψ***. With *f_αβ_*, one can write $\delta \Psi_{\alpha }=f_{\alpha \beta }*\delta \Psi_{\alpha }=\int dt_{1}f_{\alpha \beta }\left(t,t_{1}\right)\delta \Psi_{\beta }\left(t_{1}\right)$, where nonlinearities and time dependence are implicitly taken into account in the nonequilibrium *f_αβ_*:

(3) $$f_{\alpha \beta }\left(t,t^{\prime }\right)=g_{0,\alpha \beta }\left(t-t^{\prime }\right)+{\bar{\chi }}_{\alpha \beta }[\Psi ]\left(t,t^{\prime }\right),$$

(4) $${\bar{\chi }}_{\alpha \beta }[\Psi ]\left(t,t^{\prime }\right)=\pi_{\alpha \beta }\left(t,t^{\prime }\right)+\left(\frac{\delta \pi_{\alpha \beta }\left(t,t_{1}\right)}{\delta \Psi_{\beta }\left(t^{\prime }\right)}*\text{Δ}\Psi_{\beta }\left(t_{1}\right)\right)\left(t,t^{\prime }\right).$$

(More details in Supplemental Material [13].)

The connected nonequilibrium response function *F_ij_* of a general effective edge (*i*, *j*) in a network is obtained following similar steps to the ones leading to Eq. (2), but using Eq. (3) for the edge (*α*, *β*), and is

(5) $$F_{ij}\left(t,t^{\prime }\right)=G_{0,ij}^{j}\left(t-t^{\prime }\right)+\left(G_{0,i\alpha }^{j}*{\bar{\chi }}_{\alpha \beta }*F_{\beta j}\right)\left(t,t^{\prime }\right),$$

where $\left(A*B\right)\left(t,t^{\prime }\right)=\int dt_{1}A\left(t,t_{1}\right)B\left(t_{1},t^{\prime }\right)$ (note the two-times convolution [15,16]). The response *δΨ_i_* to a perturbation *δΨ_j_* can be written as a simple convolution *δΨ_i_*(*t*) = (*F_ij_* * *δΨ_j_*(*t*), where *F_ij_* evolves due to the nonequilibrium terms $\bar{\chi }\left[\Psi \right]$ and *π*[***Ψ***]. The *π*[***Ψ***] and $\bar{\chi }\left[\Psi \right]$ we consider below can be derived exactly, but there is no one recipe for calculating all possible *π*[***Ψ***] and $\bar{\chi }\left[\Psi \right]$. Condensed matter physics provides approximations and techniques for calculating them in more complicated cases [15]. Equation (5) contains different terms when *i* and/or *j* are equal to *α* and *β* (see Supplemental Material [13]).

The more the neurons on the edge (*α*, *β*) act as hubs in the network, the larger the fraction of the functional connectivity affected by their nonlinearity is. For example, *β* could be hublike because it is an interneuron integrating inputs from multiple neurons. Sensory neurons can also act as hubs. In *C. elegans*, sensory neurons can be well interconnected with the rest of the network [22]. The application of a sensory stimulus could drive (*α*, *β*) in a nonlinear regime and, therefore, alter effective interactions between other neurons. The prevalence of hubs in the brain makes our framework particularly valuable.

This framework has computational and conceptual advantages. Once the nonlinear ${\bar{\chi }}_{\alpha \beta }\left[\Psi \right]$ is calculated, *F_ij_* can be calculated for a given effective edge (*i*, *j*) via simple convolutions without needing all the details of the network: It is only necessary to run the calculation for (*i*, *j*) and (*β*, *j*). As the number of nonlinear edges increases, the last term in Eq. (5) becomes a summation over all nonlinear edges (*α*, *β*) and the computation becomes more intensive (while still limited to nonlinear edges). Overall this approach remains computationally efficient for characterizing the network, because the response functions allow one to compute the responses of the system to arbitrary stimuli.

Equations (3) and (5) explicitly describe how time-dependent approximately linear regimes arise from nonlinearities, in contrast to other, more phenomenological, locally linear methods [23,24] (see Supplemental Material [13]).

## Experimental characterization.—

Importantly, *F_ij_*’s can be obtained experimentally as responses to impulsive perturbations. The *F_ij_*’s are always well defined experimentally and theoretically, even in subsampled networks (see Experimental characterization in Supplemental Material [13]).

The local *f_ij_* are also of interest, however, because they directly relate to anatomical connections between neurons and their associated underlying molecular mechanisms. For models with equations in differential form, several approaches have been proposed to fit local parameters from spontaneous neural activity, especially in spiking neurons [25–29].

In the integral formulation, the local *f_ij_* can be obtained from measured *F_ij_* using Eqs. (2) and (5) and deconvolutions, so long as *F_ij_* is measured for each pair (*i*, *j*) and with a suitable “scan” across nonlinearities. While this is experimentally impractical for larger animals, it might be achievable in smaller ones like *C. elegans*. (De)convolutions are susceptible to noise, however, so in real measurements the response functions might need to be parametrized depending on the noise. We emphasize that, even then, Eqs. (2) and (5) are sufficient to find *f_ij_*. The ability to selectively introduce nonlinearities and the availability of fast routines to calculate the response functions will prove valuable in fits, where functions have to be evaluated several times.

## C. elegans nervous system.—

For calculations we need to provide explicit expressions of the equilibrium Green’s function and the nonlinear term *π*, beyond Eq. (5). We follow Refs. [6,7] to simulate neural dynamics in *C. elegans*. Here, the neural activity *Ψ_i_* is the membrane potential *V_i_*, and each neuron *i* is described as a single electrical compartment [6,7] via

(6) $$\partial_{t}V_{i}=-\gamma_{i}\left(V_{i}-E_{c,i}\right)-\underset{j}{\sum }\left[\gamma_{ij}^{g}\left(V_{i}-V_{j}\right)+\gamma_{ij}^{s}s_{ij}\left(V_{i}-E_{ij}\right)\right],$$

where the constants *γ* have dimensions of a conductance over a capacitance and describe leakage (*γ_i_*), electrical synapses, or gap junctions $\left(\gamma_{ij}^{g}\right)$, and chemical synapses $\left(\gamma_{ij}^{s}\right)$. *E_c,i_* is the reversal potential of the leaking channels, and *E_ij_* the reversal potential of the synaptic ionotropic receptors. *s_ij_* is a synaptic activity variable that evolves via

(7) $$\partial_{t}s_{ij}=a_{r}\phi_{ij}\left(V_{j}\right)\left(1-s_{ij}\right)-a_{d}s_{ij}.$$

*ϕ_ij_*(*V_j_*) describes the dependence of the calcium influx in the presynaptic site on the presynaptic voltage, which triggers vesicle release and is modeled as $\phi_{ij}\left(V_{j}\right)=1/\left(1+e^{-\beta_{ij}\left(V_{j}-V_{\text{th},ij}\right)}\right)$ [6,7]. Currents *I*_ext,*i*_ for external stimuli injected into neurons are added to Eq. (6) as −*I*_ext,*i*_/*C_i_*, where *C_i_* is the membrane capacitance of neuron *i*.

We obtain expressions for the equilibrium Green’s function of the system by linearizing Eqs. (6) and (7) around the equilibrium of the membrane potentials. Let Δ*V_j_* and Δ*s_ij_* be deviations from equilibrium, then we obtain $\Delta V_{i}\left(t\right)=\Sigma_{j}\left(g_{0,ij}^{g}*\Delta V_{j}\right)\left(t\right)+\left(g_{0,ij}^{s}*\Delta s_{ij}\right)\left(t\right)$ and $\Delta s_{ij}\left(t\right)=\left(\sigma_{0,ij}*\Delta V_{j}\right)\left(t\right)$. The total direct Green’s function (*V_i_* ← *V_j_* is $g_{0,ij}=g_{0,ij}^{g}+g_{0,ij}^{s}*\sigma_{0,ij}$, with $\Delta V_{i}\left(t\right)=\Sigma_{j}\left(g_{0,ij}*\Delta V_{j}\right)\left(t\right)$. (Lower case means direct. Full expressions in Supplemental Material [13].)

We obtain the full expression for the nonequilibrium *σ_ij_*[***V***](*t*, *t′*) [that yields *s_ij_*(*t*) when convolved with *V_j_*(*t*)] by reinserting the nonlinear terms in the equations,

(8) $$\sigma_{ij}\left(t,t^{\prime }\right)=\frac{\sigma_{0,ij}\left(t-t^{\prime }\right)}{\partial_{V_{j}}\phi_{ij}{|}_{\mathrm{eq}}}\frac{\text{Δ}\phi_{ij}\left(V_{j}\left(t^{\prime }\right)\right)}{\text{Δ}V_{j}\left(t^{\prime }\right)}\left[1-\frac{\left(\sigma_{ij}*\text{Δ}V_{j}\right)\left(t^{\prime }\right)}{1-s_{ij,\text{eq}}}\right],$$

where Δ*ϕ_ij_* = *ϕ_ij_* − *ϕ*_*ij*,eq_. The nonequilibrium *g_ij_*[***V***](*t*, *t′*) is

(9) $$g_{ij}\left(t,t^{\prime }\right)=g_{0,ij}^{g}\left(t-t^{\prime }\right)+g_{0,ij}^{s}*\left[\left(1-\frac{\text{Δ}V_{i}}{E_{ij}-V_{i,\text{eq}}}\right)\sigma_{ij}\right]\left(t,t^{\prime }\right).$$

The nonlinearities come from the sigmoid *ϕ* and saturation of receptors and of postsynaptic current (details and derivation in Supplemental Material [13]).

In the following examples, we only consider nonlinear contributions from *σ_α,β_*(*t*, *t′*), while we keep the equilibrium $g_{0,\alpha \beta }\left(t-t^{\prime }\right)$. The nonequilibrium response function *χ* defined by $\delta s_{\alpha \beta }\left(t\right)=\chi_{\alpha \beta }\left(t,t^{\prime }\right)*\delta V_{\beta }\left(t\right)$, with $\delta V_{\beta }$ on top of the current state $V_{\beta ,\text{eq}}+\Delta V_{\beta }$, is

(10) $$\chi_{\alpha \beta }\left(t,t^{\prime }\right)=\frac{\sigma_{0,\alpha \beta }\left(t-t^{\prime }\right)}{\partial_{V_{\beta }}\phi_{\alpha \beta }{|}_{\mathrm{eq}}}\partial_{V_{\beta }}\phi_{\alpha \beta |t^{\prime }}\left[1-\frac{\left(\sigma_{\alpha \beta }*\text{Δ}V_{\beta }\right)\left(t^{\prime }\right)}{1-s_{\alpha \beta ,\text{eq}}}\right]\;-\overset{t}{\underset{t^{\prime }}{\int }}dq\frac{\sigma_{0,\alpha \beta }(t-q)}{{\partial_{V_{\beta }}\phi_{\alpha \beta }|}_{{}_{\text{eq}}}\left(1-s_{\alpha \beta ,\text{eq}}\right)}\;\times \Delta \phi_{\alpha \beta }\left(V_{\beta }(q)\right)\chi_{\alpha \beta }\left(q,t^{\prime }\right),$$

with the direct response function $f_{\alpha \beta }=g_{0,\alpha \beta }^{s}*\chi_{\alpha \beta }$.

## Illustrative examples.—

We provide numerical examples in two simple networks where results are intuitive. In the Supplemental Material [13], we describe a more complex example, the steps involved in the calculation, and the validity of the linearization in the nonequilibrium state.

**FIG. 1.**
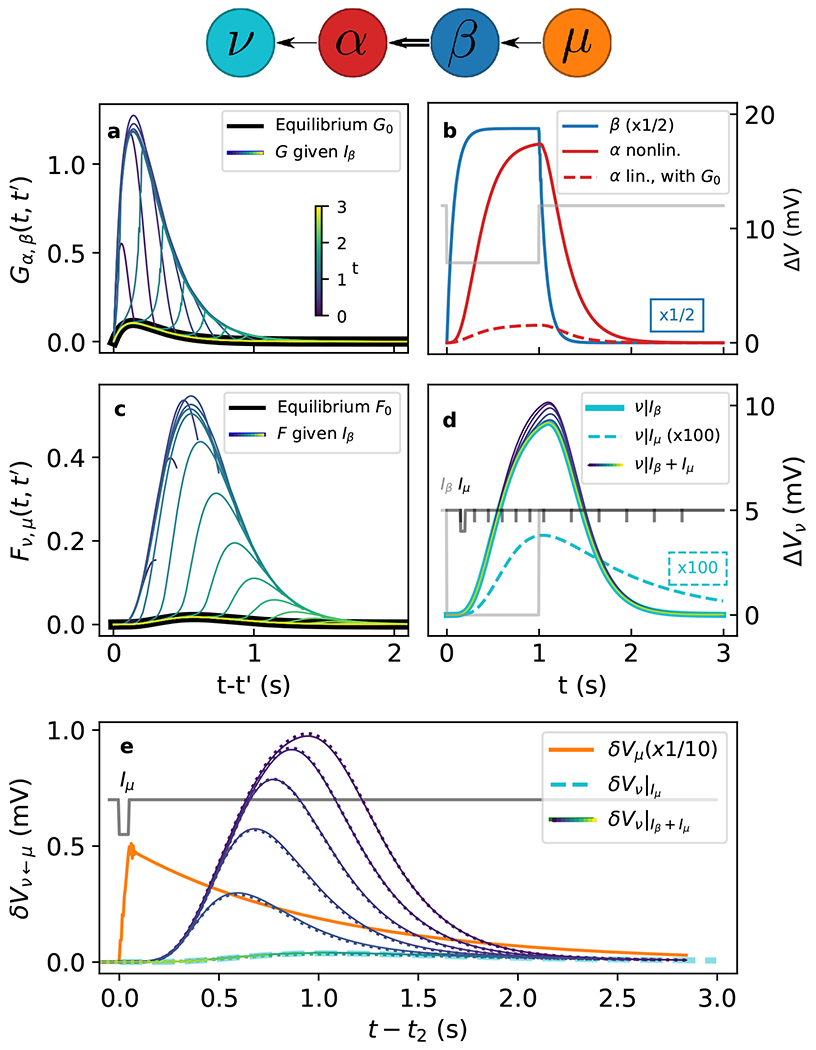
Top: Network. (a) [(c)] Nonequilibrium and equilibrium $G_{\left(0,\right)\alpha \beta }\left(t,\;t^{\prime }\right)\;\left[F_{\left(0,\right)\nu \mu }\left(t,\;t^{\prime }\right)\right]$ for selected times *t* (colored and black curves, respectively). (b) $\Delta V_{\beta }\left(t\right)$ (blue curve), and $\Delta V_{\alpha }\left(t\right)$ obtained with *G_αβ_* (solid red curve) and with *G*_0,*αβ*_ (dashed red curve). *I_β_* (gray line) with 0 baseline and −0.5 pA peak (axis not shown). (d) Δ*V_ν_* obtained with *I_β_* (solid cyan curve), *I_μ_* (dashed cyan curve, ×100), and *I_β_* + *I_μ_* [thin lines, colors as in (a)]. Gray curve as in (b). Black curve: *I_μ_*, with 0 baseline and −0.1 pA peak. Ticks: injection times *t*_2_ of *I_μ_*. (e) $\delta V_{\nu \leftarrow \mu }$ induced by *I_μ_*, calculated as $F_{\nu \mu }*\delta V_{\mu |I_{\mu }}$ (dotted lines) and as $\Delta V_{\nu |I_{\beta }+I_{\mu }}-\Delta V_{\nu |I_{\beta }}$ (solid lines), for different *t*_2_ [colors as in (a), *t*_2_ as ticks in (d)]. $\Delta V_{\nu |I_{\mu }}$ (cyan dashed line). $\delta V_{\mu |I_{\mu }}$ (orange line, ×0.1) produced by *I_μ_* (black line).

In the first example, we show how *F*(*t, t′*) correctly captures neural responses to arbitrary stimulations. The example describes a form of gating in a simple feedforward network with excitatory synaptic connections ν←α⇐β←μ, where α⇐β is the only edge where we consider a nonlinearity (as depicted in Fig. 1, top). Since we use the connected Green’s functions, the edges β←μ and ν←β can be effective representation of much larger linear subnetworks. Parameter values are similar to those in Ref. [7] (see Supplemental Material [13]), but *V*_th,*αβ*_ is set to −10 mV [30] so that *β*’s resting potential sits at the bottom of the sigmoid *ϕ_αβ_*(*V_β_*). Therefore, small perturbations around the resting potentials of neurons upstream of the nonlinear edge (*α*, *β*) produce only small responses downstream, as shown in Figs. 1(a) and 1(c), where black curves show the equilibrium $G_{0,\alpha \beta }$ and $F_{0,\nu \mu }\left(=G_{0,\nu \mu }\right)$, respectively.

The situation is different if there is a significant change of *V_β_*, e.g., if an odor stimulus is applied. To simulate this, we inject a 0.5 pA external current *I_β_* into *β* for 1 s [Fig. 1(b), gray curve], which induces a Δ*V_β_* as shown in Fig. 1(b) (blue). Consequently, *ϕ_α,β_* increases and makes *G_αβ_* transiently larger than *G*_0,*αβ*_, as Fig. 1(a) shows for selected times *t*. A larger *G_αβ_* allows the activity in *β* to reach *α* more efficiently [Fig. 1(b), solid red curve], compared to *G*_0,*αβ*_ [Fig. 1(b) dashed red curve], and consequently also other neurons downstream of edge (*α*, *β*).

While *G_αβ_* is enhanced, any other small perturbation upstream of the nonlinear edge propagates more effectively to nodes downstream, compared to at equilibrium. For example, the response function *F_νμ_* from upstream neuron *μ* to downstream neuron *ν* is shown in Fig. 1(c).

The nonequilibrium response functions obtained in the simulation via Eqs. (5) and (10) allow one to compute the response to small arbitrary perturbations without solving the underlying differential equations again (a discussion of how small is in Supplemental Material [13]). In contrast, previous methods required explicitly including any additional perturbations in the main simulation and solving the differential equations. That approach is more computationally expensive and gives less insight because the results depend on the specific perturbation, while our approach gives a characterization for any perturbation. As an illustration, we proceed both ways and compare the results.

To produce a perturbation *δV_μ_* on top of the nonequilibrium state, we consider a shorter current pulse *I_μ_* of 0.1 pA (0.05 s) injected in neuron *μ* [Fig. 1(d), black curve] at different times *t*_2_ (black ticks). The responses $\Delta V_{\nu |I_{\beta }+I_{\mu }}$ produced in neuron *ν*, explicitly calculated with both *I_β_* and *I_μ_*, are shown as thin curves in Fig. 1(d) for different *t*_2_ [same color mapping as Figs. 1(a) and 1(c)], together with $\Delta V_{\nu |I_{\beta }}$ produced by *I_β_* alone (solid cyan line). The cyan dashed line, instead, shows the $\delta V_{\nu |I_{\mu }}$ that the same perturbation would induce with the equilibrium response function *G*_0,*νμ*_, multiplied by 100.

In Fig. 1(e) we compare results obtained by explicit calculation to those obtained with the response function *F_νμ_*, aligning them in time by plotting them against *t* – *t*_2_. As a reference, the gray curve shows when *I_μ_* is applied, and the orange curve the induced *δν_μ_*. The solid lines (blue to yellow) are the responses $\delta V_{\nu \leftarrow \mu }$ due only to *δV_μ_* and calculated explicitly as $\Delta V_{\nu |I_{\beta }+I_{\mu }}-\Delta V_{\nu |I_{\beta }}$. The dotted lines are the same responses $\delta V_{\nu \leftarrow \mu }$ calculated instead using the response functions as $F_{\nu \mu }*\delta V_{\mu |I_{\mu }}$. The two calculations closely agree.

The gating effect is clear: As *I_μ_* ceases to be in coincidence with *I_β_*, its enhanced effect becomes smaller and finally vanishes when *I_μ_* is applied after *V_β_* returns to the resting value. This is also represented by the response functions in Fig. 1(c).

A second example illustrates how effective interactions can change dramatically, e.g., from an inhibitory connection to a connection that computes a fractional derivative of *δV_μ_*(*t*). We now add an inhibitory synapse *ν*|–*μ*, so that there are two paths from *μ* to *ν*, a direct inhibitory path and an indirect excitatory one through *α*⇐*β* (parameters in Supplemental Material [13]).

**FIG. 2.**
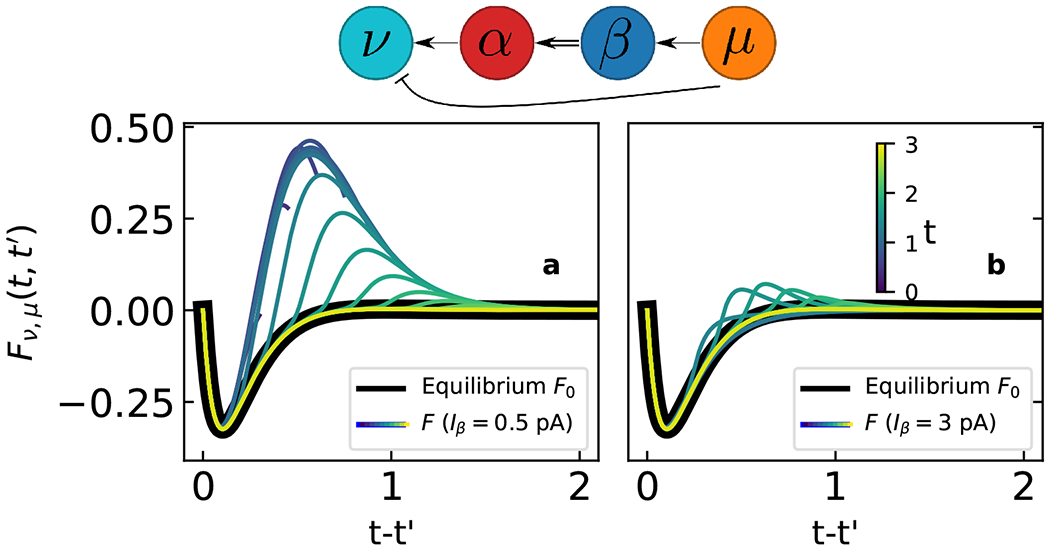
Nonequilibrium and equilibrium *F*_(0,)*νμ*_ (*t, t′*) for selected times (colored and black curves, respectively), for currents into *β* of 0.5 pA (a) and 3 pA (b). Network (top).

At equilibrium, the effective response function $F_{0,\nu \mu }\left(t-t^{\prime }\right)\left(=G_{0,\nu \mu }\right)$ is purely inhibiting [Figs. 2(a) and 2(b), black curve], because *G*_0,*αβ*_ is very small (as in the previous example). When the system is perturbed by the same square current pulse *I_β_* into neuron *β* as above, the Green’s function of the edge *α*⇐*β* is enhanced, and consequently *F_νμ_*(*t, t′*) transiently acquires the shape of a fractional derivativelike kernel shown in Fig. 2(a), before decaying back to the equilibrium *F*_0,*νμ*_.

This effect disappears if *β* is stimulated too strongly, as shown for a current of 3 pA in Fig. 2(b). As the (*α*, *β*) synapse reaches the top of *ϕ* and saturates, it becomes again unable to transmit additional perturbations. This analysis reveals how *β*’s activity influences signal propagation from *μ* to *ν* in a nontrivial way. Such a computation might exist in the brain to integrate different sensory stimuli. In our hypothetical odor example, activation of sensory neuron *β* by odorant *B* would adjust functional connectivity to modulate the animal’s downstream response to a second stimulus *M* in *μ*. Low or high concentrations of *B* would have no effect, but intermediate concentrations would cause the animal to respond to the derivative of odor *M*.

We have presented an equation for nonequilibrium Green’s functions to describe time-dependent and nonlinear networks of neurons. Two strengths of this approach stand out. First, it provides a bridge between biophysical-like models of neural networks and their effective counterparts. Second, it allows one to isolate and understand the role of specific sets of neurons in modulating functional connectivity of neural networks. This is especially valuable in *C. elegans* where the most significant nonlinearities may be localized to specific degrees of freedom or edges. We presented numerical examples that show how a nonlinear edge modifies the interaction between other neurons in a time-dependent way. We chose examples from simple networks. But, since the calculations describe the time evolution of the effective “connected” Green’s function, they hold whether the paths are direct, indirect, or involve recurrence. Therefore, the illustrated examples are representative of how hub neuron’s nonlinearities can impact large portions of the network’s functional connectivity.

## Supplementary Material

Supplementary Figure 1

Supplementary Material (creative commons license)

## References

[1] Rickgauer JP, Deisseroth K, and Tank DW, Simultaneous cellular-resolution optical perturbation and imaging of place cell firing fields, Nat. Neurosci 17, 1816 (2014). 25402854 10.1038/nn.3866 PMC4459599

[2] Emiliani V, Cohen AE, Deisseroth K, and Häusser M, All-optical interrogation of neural circuits, J. Neurosci 35, 13917 (2015). 26468193 10.1523/JNEUROSCI.2916-15.2015 PMC4604230

[3] Yang W, Carrillo-Reid L, Bando Y, Peterka DS, and Yuste R, Simultaneous two-photon imaging and two-photon optogenetics of cortical circuits in three dimensions, Elife 7, e32671 (2018). 29412138 10.7554/eLife.32671 PMC5832414

[4] Dayan P and Abbot L, Theoretical Neuroscience, Computational Neuroscience (MIT Press, Cambridge, MA, 2001).

[5] Ermentrout GB and Terman DH, Mathematical Foundations of Neuroscience, Interdisciplinary Applied Mathematics (Springer, New York, 2010).

[6] Wicks SR, Roehrig CJ, and Ranking CH, A dynamic network simulation of the nematode tap withdrawal circuit: Predictions concerning synaptic function using behavioral criteria, J. Neurosci 16, 4017 (1996). 8656295 10.1523/JNEUROSCI.16-12-04017.1996 PMC6578605

[7] Kunert J, Shlizerman E, and Kutz JN, Low-dimensional functionality of complex network dynamics: Neurosensory integration in the *Caenorhabditis elegans* connectome, Phys. Rev. E 89, 052805 (2014). 10.1103/PhysRevE.89.052805 25353842

[8] Kunert-Graf JM, Shlizerman E, Walker A, and Kutz JN, Multistability and long-timescale transients encoded by network structure in a model of *C. elegans* connectome dynamics, Front. Comput. Neurosci 11, 53 (2017). 28659783 10.3389/fncom.2017.00053 PMC5468412

[9] Kunert JM, Proctor JL, Brunton SL, and Kutz JN, Spatiotemporal feedback and network structure drive and encode *Caenorhabditis elegans* locomotion, PLoS Comput. Biol 13, e1005303 (2017). 28076347 10.1371/journal.pcbi.1005303 PMC5226684

[10] Brinkman BAW, Rieke F, Shea-Brown E, and Buice MA, Predicting how and when hidden neurons skew measured synaptic interactions, PLoS Comput. Biol 14, e1006490 (2018). 30346943 10.1371/journal.pcbi.1006490 PMC6219819

[R11] [11]Δ*Ψ_j_* is sufficient if *j* is the only neuron setting the boundary conditions. In practice, if *j* is the only neuron being externally perturbed.

[12] Linz P, Analytical and Numerical Methods for Volterra Equations, Studies in Applied and Numerical Mathematics (Society for Industrial and Applied Mathematics, 1985).

[13] See Supplemental Material at 10.1103/PhysRevLett.126.118102 for a summary of the notation, detailed derivation of the equations, and list of the parameters used in the numerical simulations.

[14] Ocker GK, Josić K, Shea-Brown E, and Buice MA, Linking structure and activity in nonlinear spiking networks, PLoS Comput. Biol 13, e1005583 (2017). 28644840 10.1371/journal.pcbi.1005583 PMC5507396

[15] Aoki H, Tsuji A, Eckstein M, Kollar M, Oka T, and Werner P, Nonequilibrium dynamical mean-field theory and its applications, Rev. Mod. Phys 86, 779 (2014).

[16] Schüler M, Golež D, Murakami Y, Bittner N, Hermann A, Strand HUR, Werner P, and Eckstein M, NESSi: The non-equilibrium systems simulation package, arXiv:1911.01211.

[17] Herrera-Delgado E, Briscoe J, and Sollich P, Tractable nonlinear memory functions as a tool to capture and explain dynamical behaviors, Phys. Rev. Research 2, 043069 (2020). 10.1103/PhysRevResearch.2.043069 PMC7614247 36855604

[18] Liu Q, Hollopeter G, and Jorgensen EM, Graded synaptic transmission at the Caenorhabditis elegans neuromuscular junction, Proc. Natl. Acad. Sci. U.S.A 106, 10823 (2009). 19528650 10.1073/pnas.0903570106 PMC2705609

[19] Lindsay TH, Thiele TR, and Lockery SR, Optogenetic analysis of synaptic transmission in the central nervous system of the nematode Caenorhabditis elegans, Nat. Commun 2, 306 (2011). 21556060 10.1038/ncomms1304 PMC3935721

[20] Narayan A, Laurent G, and Sternberg PW, Transfer characteristics of a thermosensory synapse in Caenor-habditis elegans, Proc. Natl. Acad. Sci. U.S.A 108, 9667 (2011). 21606366 10.1073/pnas.1106617108 PMC3111291

[21] Liu M, Sharma AK, Shaevitz JW, and Leifer AM, Temporal processing and context dependency in *Caenorhabditis elegans* response to mechanosensation, eLife 7, e36419 (2018). 29943731 10.7554/eLife.36419 PMC6054533

[22] Dobosiewicz M, Liu Q, and Bargman CI, Reliability of an interneuron response depends on an integrated sensory state, eLife 8, e50566 (2019). 31718773 10.7554/eLife.50566 PMC6894930

[23] Linderman S, Nichols A, Biel D, Zimmer M, and Paninski L, Hierarchical recurrent state space models reveal discrete and continuous dynamics of neural activity in C. elegans, 10.1101/621540.

[24] Costa AC, Ahamed T, and Stephens GJ, Adaptive, locally linear models of complex dynamics, Proc. Natl. Acad. Sci. U.S.A 116, 1501 (2019). 30655347 10.1073/pnas.1813476116 PMC6358715

[25] Pillow JW and Latham PE, Neural characterization in partially observed populations of spiking neurons, in Advances in Neural Information Processing Systems, edited by Platt J, Koller D, Singer Y, and Roweis S (MIT Press, Cambridge, MA, 2007), Vol. 20, pp. 1161–1168.

[26] Soudry D, Keshry S, Stinson P, Oh M, Iyengar G, and Paninski L, Efficient “Shotgun” inference of neural connectivity from highly sub-sampled activity data, PLoS Comput. Biol 11, e1004464 (2015). 26465147 10.1371/journal.pcbi.1004464 PMC4605541

[27] Dunn B and Roudi Y, Learning and inference in a non-equilibrium Ising model with hidden nodes, Phys. Rev. E 87, 022127 (2013). 10.1103/PhysRevE.87.022127 23496479

[28] Tyrcha J and Hertz J, Network inference with hidden units, Math. Biosci. Eng 11, 149 (2014). 24245678 10.3934/mbe.2014.11.149

[29] Bravi P, Opper M, and Sollich P, Inferring hidden states in Langevin dynamics on large networks: Average case performance, Phys. Rev. E 95, 012122 (2017). 28208380 10.1103/PhysRevE.95.012122

[30] Juusola M, French AS, Uusitalo RO, and Weckström M, Information processing by graded-potential transmission through tonically active synapses, Trends Neurosci. 19, 292 (1996). 8799975 10.1016/S0166-2236(96)10028-X

