# Supplementary figures and images for: Nonequilibrium Green’s Functions for Functional Connectivity in the Brain
Source: Phys Rev Lett. Author manuscript; Available in PMC 2021 Sep 21. (PMC8454901; doi:10.1103/PhysRevLett.126.118102)

**a**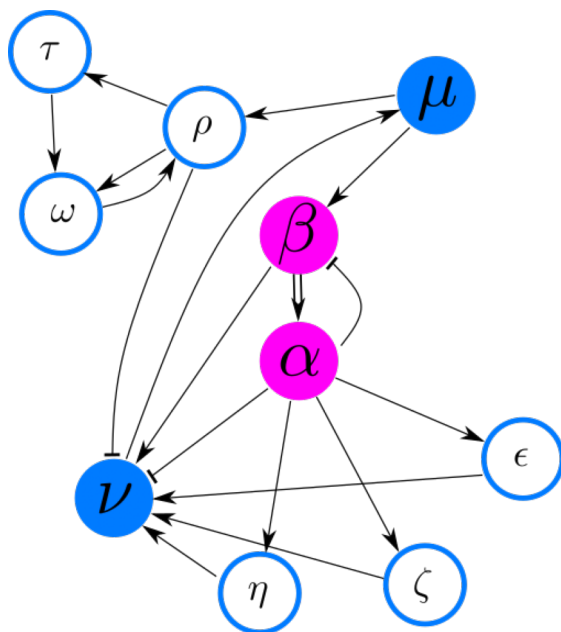**b**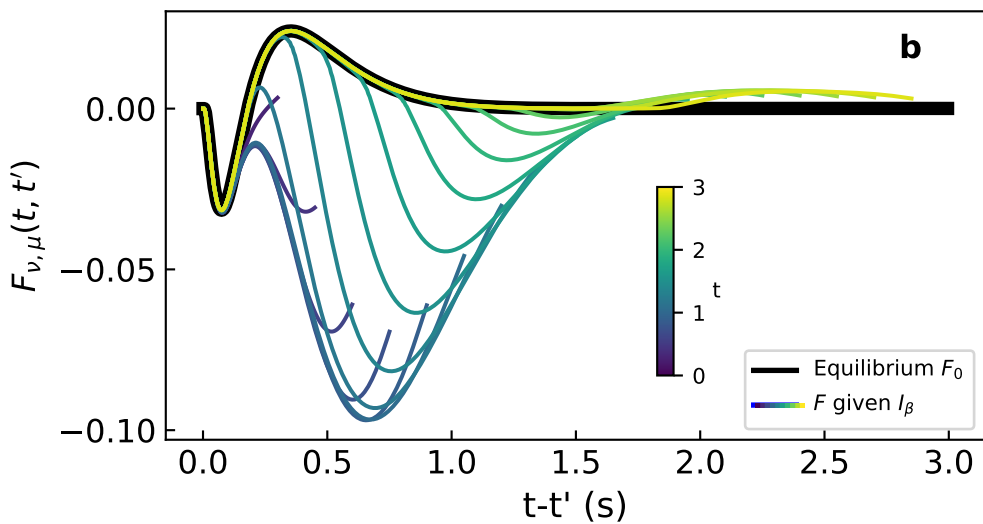**c**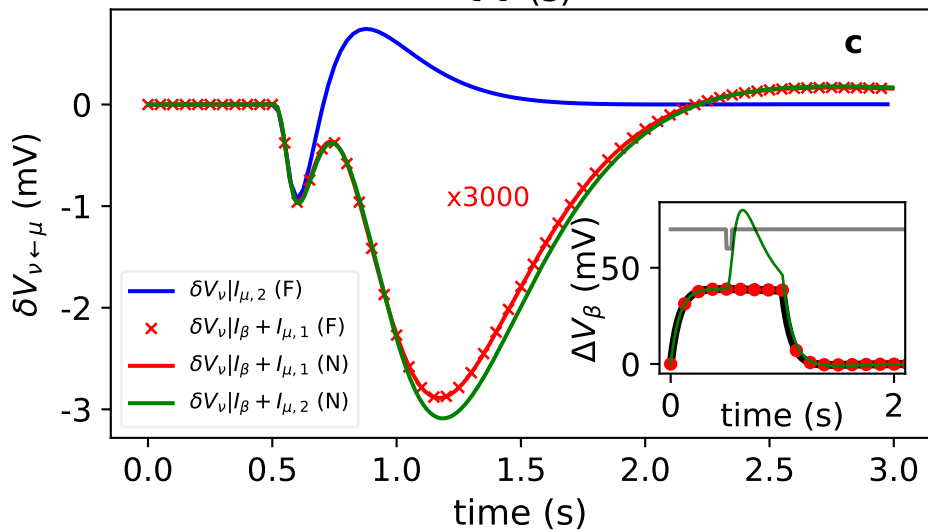

Supplement: Supplementary Figure 1 [file NIHMS1740445-supplement-Supplementary_Figure_1.pdf]
