# Supplementary material for: Nonequilibrium Green’s Functions for Functional Connectivity in the Brain
Source: Phys Rev Lett. Author manuscript; Available in PMC 2021 Sep 21. (PMC8454901; doi:10.1103/PhysRevLett.126.118102)
Supplement: Supplementary Material (creative commons license) [file NIHMS1740445-supplement-Supplementary_Material__creative_commons_license_.pdf]

Supplemental material for: Nonequilibrium Green's functions for functional connectivity in the brain

Francesco Randi[1] and Andrew M. Leifer[1,2]

[1]*Department of Physics, Princeton University, Jadwin Hall, Princeton, NJ 08544, USA*
[2]*Princeton Neuroscience Institute, Princeton University, NJ 08544, USA*

(Dated: February 18, 2021)

A theoretical framework describing the set of interactions between neurons in the brain, or functional connectivity, should include dynamical functions representing the propagation of signal from one neuron to another. Green's functions and response functions are natural candidates for this but, while they are conceptually very useful, they are usually defined only for linear time-translationally invariant systems. The brain, instead, behaves nonlinearly and in a time-dependent way. Here, we use nonequilibrium Green's functions to describe the time-dependent functional connectivity of a continuous-variable network of neurons. We show how the connectivity is related to the measurable response functions, and provide two illustrative examples via numerical calculations, inspired from *C. elegans*.

NOTATION

g is for Green's functions, f is for response functions. Lower case is for the direct paths, upper case for the effective ones. Subscript 0 stands for time-translationally invariant. The superscript on G_0 denotes the indices that are excluded from the summation in the definition of G_0.

Green's functions:

- $g_{0,ij}(t-t')$: equilibrium direct Green's function for the pair of neurons $i \leftarrow j$
- $G^{j}_{0,ij}(t-t')$: equilibrium effective Green's function for neurons $i \leftarrow j$ in a network
- $g_{ij}(t,t') = g_{0,ij}(t-t') + \pi_{ij}[\boldsymbol{\psi}](t,t')$: nonequilibrium direct Green's function written as the sum of the equilibrium $g_{0,ij}$ and a nonequilibrium term π_{ij} that depends on the state $\boldsymbol{\psi}$ of the system.
- $G_{ij}(t,t')$: nonequilibrium effective Green's functions for neurons in a network

Response functions

- $f_{0,ij}(t-t')$: equilibrium direct response function for an isolated pair of neurons (equal to $g_{0,ij}$)
- $F_{0,ij}(t-t')$: equilibrium effective response function for two neurons in a network (equal to $G^{j}_{0,ij}$)
- $f_{ij}(t,t') = f_{0,ij}(t-t') + \overline{\chi}_{ij}[\boldsymbol{\psi}](t,t')$: nonequilibrium direct response function for an isolated pair of neurons, written as the sum of the equilibrium response function $f_{0,ij}$ and a nonequilibrium term $\overline{\chi}_{ij}$ which depends on the state $\boldsymbol{\psi}$ of the system.
- $F_{ij}(t,t')$: nonequilibrium response function for neurons in a network

LINEAR AND TIME-TRANSLATIONALLY INVARIANT CASE

Isolated pair of neurons

In the linear and time-translationally invariant (TTI) case, the activity $\psi_i(t) = \psi_{i,\mathrm{eq}} + \Delta\psi_i(t)$ of neuron i is given by

$$\psi_i(t) = \psi_{i,eq} + \sum_{j\in\ \mathrm{all}} g_{0,ij} * \Delta\psi_j\,(t) + g^{\mathrm{ext}}_{0,i} * I^{\mathrm{ext}}_i(t), \quad (1)$$

where the ψ_{eq} are the resting activity levels of the neurons (which depend on the equilibrium inputs from the other neurons) and the $\Delta\psi$ are the changes of the activity with respect to those resting values. $*$ denotes a convolution such that

$$\begin{aligned}\big(g * \Delta\psi\big)(t) &= \int dt_1\, g(t,t_1)\Delta\psi(t_1) \\ g(t,t') &= g(t-t') \text{ for TTI case}\end{aligned} \quad (2)$$

$g_{0,ij}(t-t')$ is the Green's function, or transfer function, describing the direct interaction $i \leftarrow j$ from neuron j to neuron i (the subscript 0 denotes their time-translational invariance). I^{ext}_i is an external input to j, and $g^{\mathrm{ext}}_{0,i}$ is the transfer function needed to calculate the activity induced by I^{ext}_i. Note that we use $\Delta\psi$ to denote the deviation of the current state from the equilibrium activity, and $\delta\psi$ to denote perturbations induced in order to probe the properties of the system (see below).

If we consider an isolated pair of neurons i and j, not connected to any network and with a one-way connection $i \leftarrow j$, then the right-hand side of Eq. (1) for neuron i contains a convolution only for the single neuron j. If we know $\Delta\psi_j$, and $\Delta\psi_j$ entirely sets the boundary conditions of the problem because, e.g. it is the only neuron being perturbed, the $\psi_i(t)$ calculated from Eq. (1) is exact. In this situation, j would not have any input but I^{ext}_j, and would be $\Delta\psi_j(t) = \psi_{j,\mathrm{eq}} + g^{\mathrm{ext}}_{0,j} * I^{\mathrm{ext}}_j(t)$

A strategy to probe the dynamical properties of the two-neurons system in a given state $\boldsymbol{\psi} = \boldsymbol{\psi}_{\mathbf{eq}} + \boldsymbol{\Delta\psi}$ is to measure its response functions. $\boldsymbol{\Delta\psi}$, here, has been produced in an unspecified way: for example by an external perturbation, a sensory stimulus, or a change in the cellular properties that has determined a change in the resting activity of the neuron with respect to the usual equilibrium. To measure the response function, we can induce in j an additional perturbation $\delta\psi_j$ via an external current I_j^{ext} and measure the response that this produces in i. This response can be expressed as

$$\delta\psi_i(t) = f_{0,ij} * \delta\psi_j\,(t). \tag{3}$$

$f_{0,ij}(t-t')$ is the response function of the edge (i,j), and in the linear and time-translationally invariant case is equal to the Green's function $g_{0,ij}(t-t')$. Moreover, since the system is linear, Eq. (3) is valid for $\delta\psi$s of any magnitude.

Neurons in a network

Eq. (1) only describes direct connections between the neurons. If the pair (i,j) is embedded in a network, the signal can travel from j to i both on direct and indirect paths. Therefore, if the boundary conditions are set by $\Delta\psi_j$ as above, in this case Eq. (1) yields a result that is correct only up to the first perturbative order. To obtain the exact result, Eq. (1) should be solved for each neuron in the network, and the values of $\psi(t)$ updated at each time-step t for each neuron.

However, the direct Green's functions $g_{0,ij}$ of a linear system can be condensed in effective, or connected, Green's functions $G^j_{0,ij}(t-t')$, which in the context of Volterra integral equations are the so-called resolvent kernels [1] (for the meaning of the superscript j see below). If we know $\Delta\psi_j$ and if we want to calculate the activity $\Delta\psi_i$ produced in i without calculating $\Delta\psi$ for the whole network (and if the boundary conditions are set by $\Delta\psi_j$), we can recursively insert the contribution from all the neurons (and therefore all the paths) in Eq. (1) to obtain such connected Green's function. The goal is to write

$$\psi_i(t) = \psi_{i,\text{eq}} + G^j_{0,ij} * \Delta\psi_j(t). \tag{4}$$

Focusing on the $\Delta\psi_i$ in Eq. (1), dropping for the moment the external inputs, we have

$$\Delta\psi_i(t) = g_{0,ij} * \Delta\psi_j + \sum_{\mu\neq j} g_{0,i\mu} * \Delta\psi_\mu. \tag{5}$$

The $\Delta\psi_\mu$s are themselves only determined by $\Delta\psi_j$, so that we can substitute $\Delta\psi_\mu$ with $g_{0,\mu j} * \Delta\psi_j + \sum_\nu g_{0,\mu\nu} * \Delta\psi_\nu$

$$\begin{aligned}\Delta\psi_i(t) = g_{0,ij} * \Delta\psi_j + \sum_{\mu\neq j} g_{0,i\mu} * g_{0,\mu j} * \Delta\psi_j \\ + \sum_{\mu\neq j}\sum_{\nu} g_{0,i\mu} * g_{0,\mu\nu} * \Delta\psi_\nu.\end{aligned} \tag{6}$$

Recursively repeating this step, we obtain

$$\begin{aligned}\Delta\psi_i(t) = \Bigg\{ g_{0,ij} + \sum_{\mu\neq j} g_{0,i\mu} * \\ \Bigg[g_{0,\mu j} + \sum_{\nu\neq j} g_{0,\mu\nu} * g_{0,\nu j} + \ldots \Bigg]\Bigg\} * \Delta\psi_j\,(t).\end{aligned} \tag{7}$$

$G^j_{0,ij}$ can be read off the above equation comparing it with Eq. (4). The superscript j denotes the fact that j is excluded in the sums.

Additionally, noting that the terms inside the square brackets are $G^j_{0,\mu j}$, we obtain an equation relating the direct and connected Green's functions:

$$G^j_{0,ij} = g_{0,ij} + \sum_{\mu\neq j} g_{0,i\mu} * G^j_{0,\mu j}. \tag{8}$$

In a linear system, one would write the above equation in Fourier space, where the convolutions are transformed in simple products, like in Ref. [2]. But this cannot be done when the functions are not time-translationally invariant, as is the case in the main discussion of this work.

Because we already take into account indirect paths via G_0, in Eq. (4) the convolution is summed only over the neurons that set the boundary conditions. In the simple example in which an external current is injected in a single neuron j, the convolution should be performed only with $\Delta\psi_j$. If an external current is injected in two neurons, the convolutions in Eq. (4) should be with the activities produced by those external currents separately.

As in the previous section, we can characterize the system measuring its response functions. This can be done experimentally by inducing a perturbation $\delta\psi_j(t)$ and measuring the produced $\delta\psi_i(t)$. Also for neurons in a network, in the linear and time-translationally invariant case the response functions $F_{0,ij}$ are equal to the Green's functions $G^j_{0,ij}$, such that one can write

$$\delta\psi_i(t) = F_{0,ij} * \delta\psi_j\,(t) \tag{9}$$

The activity produced by an external current I_j^{ext} in j also contains a network term if the neurons are in a network. With a recursive procedure similar to the one leading to Eq. (8), one obtains that the $\Delta\psi_j$ induced by I_j^{ext} is

$$\Delta\psi_j(t) = (\delta(t,t') + G_{0,jj}) * g^{\text{ext}}_{0,j} * I^{\text{ext}}_j. \tag{10}$$

$G_{0,jj}$ does not have a superscript j, which means that it should be calculated as

$$G_{0,jj} = g_{0,jj} + \sum_{\mu\in\text{ all}} g_{0,j\mu} * G_{0,\mu j}. \tag{11}$$

The next section explains why in some cases some indices have to be excluded from the sums and in others they don't.

Self loops, *measured* activities, and alternative formulation

The goal of writing $\Delta\psi_i = G^j_{0,ij} * \Delta\psi_j$, and $\delta\psi_i = F_{0,ij} * \delta\psi_j$, is to have expressions that relate *measured* activities. If we know $\Delta\psi_j(t)$ because we measured it, $\Delta\psi_j$ already contains all the corrections due to the presence of the network. In the summation in Eq. (8), μ cannot be equal to j because otherwise we would be considering paths that start from j and return to j before coming to i. These paths affect $\Delta\psi_j$ because they bring input to j, so their contribution has already been accounted for in the known $\Delta\psi_j$. Excluding j from the summation in Eq. (8) is equivalent to considering paths that start from j and end on i without ever coming back to j.

When we want to calculate, instead, the activity $\Delta\psi_j$ produced by an external current injected into j (Eq. (10)), we need to consider those network effects on j itself that we discarded above. This is why in Eq. (10), $G_{0,jj}$ appears without a superscript j.

Alternative formulation An alternative formulation in which no index is excluded from all $G_{0,ij}$s is possible, but comes with a crucial disadvantage. Let us call $\Delta\overline{\psi}_j(t)$ the activity that an external stimulation I^{ext}_j would produce in neuron j if it were *isolated*

$$\Delta\overline{\psi}_j(t) = g^{\text{ext}}_{0,j} * I^{\text{ext}}_j. \tag{12}$$

The activity of that same neuron j considering the feedback from the network, would be given by $\Delta\psi_j = (\delta(t,t') + G_{0,jj}) * \Delta\overline{\psi}_j$ (the same as Eq. (10)). The activity of other neurons i due to $\Delta\psi_j$ would now be given by $\Delta\psi_i(t) = G_{0,ij} * \Delta\overline{\psi}_j(t)$, with the summation in $G_{0,ij}$ running over all indices.

However, the relation now is between the measured $\Delta\psi_i$ and $\Delta\overline{\psi}_j$, the activity of neuron j if it were isolated from the network, which cannot be measured. To make use of this relation to obtain information about the network, we need to make assumptions about $\Delta\overline{\psi}_j$, which translate into assumptions about $g^{\text{ext}}_{0,j}$ and I^{ext}_j. In some cases, g^{ext} and I^{ext} can be estimated or separately characterized, like in the case of optogenetic stimulations with light-gated ion channels, or channelrhodopsins (I^{ext} depends, for example, on the gating kinetics of the channelrhodopsin). Given that the stimulus is an electrical current, the coupling to the membrane potential of a neuron is via a first-order equation and $g^{\text{ext}} \propto e^{-t/\tau}$.

This characterization is not general, however, because in the same nervous system stimuli might be applied to the neurons also via other types of molecules, like G-protein coupled receptors. With these receptors, the coupling to the neuron's activity is likely to be at least via a second-order equation because there are intermediate steps involved, so that g^{ext} is not a simple exponential anymore.

Depending on the context and the goal of the calculations, be they for numerical simulations or data analysis, either of the two formulations (with $\Delta\overline{\psi}$ or $\Delta\psi$) can be used. In the following, we will keep working with the *measured* $\Delta\psi$.

NONEQUILIBRIUM CASE

Isolated pair of neurons

Also in the nonlinear case we would like to formally write the state of the system as a convolution of a Green's function and the past activities of the neurons:

$$\Delta\psi_i(t) = \int dt'\, g_{ij}(t,t')\Delta\psi_j(t') = g_{ij} * \Delta\psi_j\,(t). \tag{13}$$

In contrast to the linear and time-translationally invariant case, the Green's function g_{ij} in Eq. (13) is not a time-invariant kernel (hence, nonequilibrium). Instead it is, in principle, functionally dependent on the whole state of the system $\boldsymbol{\psi}$. We can write $g_{ij}[\boldsymbol{\psi}]$ as the sum of a linear and time-translationally invariant term $g_{0,ij}(t-t')$ and a nonequilibrium term $\pi_{ij}(t,t')$:

$$g_{ij}[\boldsymbol{\psi}](t,t') = g_{0,ij}(t-t') + \pi_{ij}[\boldsymbol{\psi}](t,t'). \tag{14}$$

There is no general recipe for calculating $\pi_{ij}[\boldsymbol{\psi}]$. Different models describing the nonlinear or time-dependent nature of the edge (i,j) will involve their own specific solutions. However, Eq. (13) and (14) are useful as formal devices because they will allow us to express how a given $\pi_{ij}[\boldsymbol{\psi}]$, once it is known, should be used to calculate the modified properties of the rest of the network.

Response function of isolated pair

In the nonequilibrium case, the response function f_{ij} is, in general, not equal to the Green's function g_{ij}. If the system is in the state $\boldsymbol{\psi} = \boldsymbol{\psi}_{\mathbf{eq}} + \boldsymbol{\Delta\psi}$ (where $\boldsymbol{\Delta\psi}$ has been produced in an unspecified way), and we induce a perturbation $\delta\psi_\beta$ in β to probe the system, the activity $\delta\psi_\alpha$ produced in α can still be written as

$$\delta\psi_\alpha(t) = f_{\alpha\beta} * \delta\psi_\beta\,(t), \tag{15}$$

but here $f_{\alpha\beta}$ is given by

$$f_{\alpha\beta}[\boldsymbol{\psi}](t,t') = \frac{\delta\psi_\alpha}{\delta\psi_\beta} = g_{0,\alpha\beta}(t-t') + \overline{\chi}_{\alpha\beta}[\boldsymbol{\psi}](t,t'), \tag{16}$$

where $\overline{\chi}_{\alpha\beta}[\boldsymbol{\psi}]$ is the nonequilibrium part of the response function and is given by

$$\overline{\chi}_{\alpha\beta}[\boldsymbol{\psi}](t,t') = \pi_{\alpha\beta}(t,t') + \left(\frac{\delta\pi(t,t_1)}{\delta\psi_\beta(t')} * \Delta\psi_\beta(t_1)\right)(t,t'). \tag{17}$$

Also here, f_{ij} is not a time-translationally invariant transfer function, and depends on the state of the system $\boldsymbol{\psi}$ via $\overline{\chi}_{\alpha\beta}[\boldsymbol{\psi}]$. Therefore, it has to be calculated according to its full expressions (or approximations to it). But, provided that $\delta\psi_j$ is "small" (see below), once $\overline{\chi}_{\alpha\beta}$ and f_{ij} are computed, Eq. (15) can be used to to compute the response $\delta\psi_\alpha$ to any perturbation $\delta\psi_\beta$.

In neurons, we can expect the linear-response regime to be valid also for $\delta\psi$ of significant magnitude. In the most general nonlinear system, linear responses around an arbitrary state can be very limited approximations. But nonlinearities like sigmoidal thresholds at the synapses have large activity intervals where they are well-approximated by a linear function. In the examples in the main text, at equilibrium the synapse (α,β) "sits" at the bottom of $\phi(V_\beta)$, the sigmoidal function describing the dependence of the synaptic vesicle release on the calcium influx into the presynaptic site. As the system is brought out of equilibrium, the synapse reaches the central region of the sigmoid $\phi(V_\beta)$, where the sigmoid is again almost linear and where the linear-response approximation works well.

Two cases can be discussed starting from Eq. (17). $\pi(t,t')$ can be nonequilibrium in two ways: it can be time-dependent or explicitly nonlinear (or a combination of the two). If π is purely time-dependent and does not depend on ψ_β, the only nonzero term in the right-hand side of Eq. (17) is π. In this case, the response function is equal to the Green's function: $f_{\alpha\beta} = g_{0,\alpha\beta} + \pi_{\alpha\beta}$. If π depends on $\delta\psi_\beta$, then also the second term is nonzero and the $f_{\alpha\beta} \neq g_{\alpha\beta}$.

Neurons in a network: nonequilibrium Green's functions

The connected Green's functions for neurons in a network will be determined also by the nonequilibrium terms. To derive an equation for the nonequilibrium Green's functions G_{ij} we start from the following experimental consideration related to *C. elegans* that can be taken as a general starting point. Characterizations of some synpases in the worm have shown that they are linear throughout a large part of the physiological range of membrane potentials [3–5]. However, we know that nonlinearities and time-dependences are critically important in the *C. elegans* nervous system and in nervous systems generally, because they allow the network to perform computations, including for example responding to sensory stimuli in a context dependent manner [6, 7]. How does a network have many linear edges but also show widespread nonlinear behaviors? In the integral formulation with nonequilibrium Green's functions it is straightforward to show how these two observations can coexist.

We, therefore, consider a network in which only one of the edges, (α,β), displays a significant nonlinearity. This is in contrast to an approach in [8] which assumes nonlinearities that are homogeneous over the network and then proceeds with their systematic expansion.

The connected nonequilibrium Green's function for the effective edge (i,j) can be calculated with a recursive procedure similar to the one leading from Eq. (1) to Eqs. (4) and (8), but using the nonequilibrium Green's function in Eq. (14) for the edge (α,β). The result for $i \neq \alpha, j \neq \beta$ is

$$G_{ij}(t,t') = G_{0,ij}(t-t') + G^{j}_{0,i\alpha} * \pi_{\alpha\beta}[\boldsymbol{\psi}] * G_{\beta j}\,(t,t'), \tag{18}$$

where $(A*B)(t,t') = \int dt_1\, A(t,t_1)B(t_1,t)$ and the activity $\Delta\psi_i$ can be written as

$$\Delta\psi_i(t) = \sum_{\text{b.c.}} G_{ij} * \Delta\psi_j, \tag{19}$$

with, as usual, a summation on the $\Delta\psi$s that set the boundary conditions (b.c.). Differently from Eq. (8), here G_{ij} is not a time-translationally invariant transfer function. The nonequilibrium $\pi_{\alpha\beta}[\boldsymbol{\psi}]$ and G_{ij} have to be calculated for each time-step taking the nonlinear and time-dependent nature of $\pi_{\alpha\beta}[\boldsymbol{\psi}]$ into account, and the calculation depends, therefore, on the specific state $\boldsymbol{\psi}$ considered. Once it is calculated, however, it can be plugged in in the expression for all the nonequilibrium G_{ij} (Eq. (18)), and can be used to calculate $\Delta\psi_i$ via convolutions only, without running a nonlinear calculation.

The full nonlinear calculation of $\pi_{\alpha\beta}[\boldsymbol{\psi}]$ does not involve, however, all the neurons of the network, in common situations. $\pi_{\alpha\beta}[\boldsymbol{\psi}]$ will depend likely on a limited subsets of neurons only. A nonlinearity determined by threshold or saturation at a synapse will depend only on the presynaptic activity ψ_β. If neuromodulation is involved, it will be determined by a defined set of neurons.

Additionally, in the calculation of G_{ij} the effect of the network recurrence is condensed in only a single additional effective edge (β,j). To calculate G_{ij} one needs, therefore, to simultaneously calculate only $G_{\beta,j}$.

There are some special cases for Eq. (18).

If $(i,j) = (\alpha,\beta)$

$$G_{\alpha\beta} = G^{\beta}_{0,\alpha\beta} + (\delta(t,t') + G^{\beta}_{0,\alpha\alpha}) * \pi_{\alpha\beta}. \tag{20}$$

If $i = \alpha$ and $j \neq \alpha, \beta$

$$G_{\alpha j} = G^{j}_{0,\alpha j} + (\delta(t,t') + G^{j}_{0,\alpha\alpha}) * \pi_{\alpha\beta} * G_{\beta j}. \tag{21}$$

If $i,j = \alpha$

$$G_{\alpha\alpha} = G_{0,\alpha\alpha} + (\delta(t,t') + G_{0,\alpha\alpha}) * \pi_{\alpha\beta} * G_{\beta\alpha}. \tag{22}$$

If $i \neq \alpha, \beta$ and $j = \beta$

$$G_{i\beta} = G^{\beta}_{0,i\beta} + G^{\beta}_{0,i\alpha} * \pi_{\alpha\beta}. \tag{23}$$

If $i, j = \beta$

$$G_{\beta\beta} = G_{0\beta\beta} + G_{0,\beta\alpha} * \pi_{\alpha\beta} * (\delta(t,t') + G_{\beta\beta}). \tag{24}$$

If $i \neq \alpha$ and $j = \alpha$

$$G_{i\alpha} = G^{\alpha}_{0,i\alpha}. \tag{25}$$

If $i = j$ and $i \neq \alpha, \beta$

$$G_{ii} = G_{0,ii} + G_{0,i\alpha} * \pi_{\alpha\beta} * G_{\beta i}. \tag{26}$$

Neurons in a network: nonequilibrium response functions

The response function of an effective edge (i, j) is modified by the network in a similar way as the nonequilibrium G_{ij} in Eq. (18). We assume again one single nonequilibrium edge (α, β) and insert recursively the contributions from all the neurons in Eq. (3), using Eq. (16) for the edge (α, β). The result for $i \neq \alpha, j \neq \beta$ is

$$F_{ij}(t,t') = G^{j}_{0,ij}(t-t') + \left(G^{j}_{0,i\alpha} * \overline{\chi}_{\alpha\beta} * F_{\beta j}\right)(t,t'). \tag{27}$$

The response $\delta\psi_i$ in i produced by a small perturbation $\delta\psi_j$ in j can be written as

$$\delta\psi_i(t) = \int dt'\, F_{ij}(t,t')\delta\psi_j(t'). \tag{28}$$

Also here F_{ij} is not a time-translationally invariant transfer function, and depends on the state of the system $\boldsymbol{\psi}$ via $\overline{\chi}_{\alpha\beta}[\boldsymbol{\psi}]$. But, provided that $\delta\psi_j$ is "small" (see discussion in the section about the isolated-pair response function), once $\overline{\chi}_{\alpha\beta}$ and F_{ij} are computed in the full nonlinear calculation, Eq. (28) can be used to to compute the response $\delta\psi_i$ to any perturbation $\delta\psi_j$.

Also for the response functions F_{ij}, as for the Green's functions G_{ij}, there are some special cases of Eq. (27).

If $(i, j) = (\alpha, \beta)$

$$F_{\alpha\beta} = G^{\beta}_{0,\alpha\beta} + (\delta(t,t') + G^{\beta}_{0,\alpha\alpha}) * \overline{\chi}_{\alpha\beta}. \tag{29}$$

If $i = \alpha$ and $j \neq \alpha, \beta$

$$F_{\alpha j} = G^{j}_{0,\alpha j} + (\delta(t,t') + G^{j}_{0,\alpha\alpha}) * \overline{\chi}_{\alpha\beta} * F_{\beta j}. \tag{30}$$

If $i, j = \alpha$

$$F_{\alpha\alpha} = G_{0,\alpha\alpha} + (\delta(t,t') + G_{0,\alpha\alpha}) * \overline{\chi}_{\alpha\beta} * F_{\beta\alpha}. \tag{31}$$

If $i \neq \alpha, \beta$ and $j = \beta$

$$F_{i\beta} = G^{\beta}_{0,i\beta} + G^{\beta}_{0,i\alpha} * \overline{\chi}_{\alpha\beta}. \tag{32}$$

If $i, j = \beta$

$$F_{\beta\beta} = G_{0\beta\beta} + G_{0,\beta\alpha} * \overline{\chi}_{\alpha\beta} * (\delta(t,t') + F_{\beta\beta}). \tag{33}$$

If $i \neq \alpha$ and $j = \alpha$

$$F_{i\alpha} = G^{\alpha}_{0,i\alpha}. \tag{34}$$

If $i = j$ and $i \neq \alpha, \beta$

$$F_{ii} = G_{0,ii} + G_{0,i\alpha} * \overline{\chi}_{\alpha\beta} * F_{\beta i}. \tag{35}$$

Experimental characterization

F_{ij} are the response functions that can be obtained in experiments on networks of neuron. Importantly, they are always well defined, for two reasons. First, regardless of how nonlinear the system is, an experiment can always be designed to probe the system by perturbing it and measuring its response. Second, their role is always clear even if we are not considering any other neuron in addition to i and j, both in an experiment and in theoretical calculations, even if in the latter the response function would be difficult to calculate without making assumptions on the rest of the network.

In an experiment, F_{ij} can be measured as responses to impulsive perturbations. One could object that, by applying an impulsive external stimulation I^{ext}_j to neuron j, the $\delta\psi_j$ induced in j would be given by Eq. (10) and would not be itself a delta function. Therefore, the response of neuron i would be given not by F_{ij} alone, but by the convolution of $(1 + G_{0,jj}) * g^{\text{ext}}_{0,j}$ with F_{ij}. However, if the coupling between the external stimulation I^{ext}_j and ψ_j is via a first-order differential equation, e.g. for current in an equation for membrane potential, for which $g^{\text{ext}}_{0,j} \propto e^{-(t-t')/\tau}$, this limit is of no concern. The timescale τ is a property of neuron j and applies to any input to that neuron, so that there are no response functions with a path through j that are "faster" than g^{ext}_j.

RELATION OF NONEQUILIBRIUM GREEN'S FUNCTIONS TO OTHER METHODS

Eqs. (16) and (27) (corresponding to Eqs. 2 and 4 of the main text) describe an approximately linear regime on top of an arbitrary state of the system. Switching linear dynamical systems (SLDS) models [9, 10] assume that such locally linear regimes exist. They describe the dynamics of a nonlinear system as a temporal sequence of linear systems each with different parameters, and have been applied to *C. elegans*. In existing SLDS models, the time-dependent switching between parameters is entirely phenomenological. In our approach, Eqs. (16) (main text Eq. 2), (27) (main text Eq. 4), and (18), explicitly

govern how nonlinearities in the network produce time-dependent changes to a linear system. Here the response functions contain the time-dependent parameters of the SLDS.

To draw a parallel between the numerical examples in the main text and SLDS [9, 10], the state induced by the perturbation I_μ corresponds to switching from the equilibrium linear system to another one with different parameters.

C. ELEGANS

Equilibrium Green's functions

Defining

$$\bar{a}_{ij} \equiv a_{d,ij} + a_{r,ij}\phi_{ij}(V_{j,\mathrm{eq}}) \tag{36}$$

and

$$\bar{\gamma}_i \equiv \gamma_i + \sum_j \gamma^{\mathrm{g}}_{ij} + \sum_j \gamma^{\mathrm{s}}_{ij} s_{ij,\mathrm{eq}} \tag{37}$$

the equilibrium Green's functions $\sigma_{0,ij}$, $g^{\mathrm{g}}_{0,ij}$, and $g^{\mathrm{s}}_{0,ij}$ are

$$\sigma_{0,ij}(t) = \theta(t) a_r (1 - s_{ij,\mathrm{eq}})(\partial_{V_j}\phi_{ij}\big|_{\mathrm{eq}}) e^{-\bar{a}_{ij}t}, \tag{38}$$

$$g^{\mathrm{g}}_{0,ij}(t) = \theta(t)\gamma^{\mathrm{g}}_{ij} e^{-\bar{\gamma}_i t}, \tag{39}$$

$$g^{\mathrm{s}}_{0,ij}(t) = \theta(t)\gamma^{\mathrm{s}}_{ij}(E_{ij} - V_{i,\mathrm{eq}}) e^{-\bar{\gamma}_i t}, \tag{40}$$

where $\theta(t)$ is the Heaviside step function.

Nonequilibrium Green's functions and nonlinear terms

There are three sources of nonlinearities that can be added back to the above linear and TTI Green's functions. The first is the saturation of the postsynaptic current when the postsynaptic membrane potential approaches the reversal potential of the ionotropic receptor through which the current flows (Eq. 5 of the main text). This nonlinearity changes g^{s}_{ij}. The second is the saturation of the synaptic activity (which can range between 0 and 1) due to the finite number of receptors, and the third is the sigmoidal dependence $\phi(V_j)$ of the vescicle release on the presynaptic potential (Eq. 6 of the main text). These two nonlinearities change σ_{ij} and are the ones considered in the numerical examples.

Derivation of NEGF To derive the explicit expression for the nonequilibrium Green's functions we can proceed as follows. As a reminder, in Eq. 6 of the main text electrical synapses linearly couple $V_i \leftarrow V_j$ via the equilibrium Green's function g^{g}_{ij} in Eq. (39). Chemical synapses, instead, couple $V_i \leftarrow V_j$ via an intermediate synaptic activity variable s_{ij}, governed by Eq. 7 (main text). Both the steps $V_i \leftarrow s_{ij}$ and $s_{ij} \leftarrow V_j$ involve nonlinear terms, so we derive two separate nonequilibrium Green's functions for the two steps that can be then combined, $\sigma_{ij}(t,t')$ and $g^{\mathrm{s}}_{ij}(t,t')$, that account for different nonlinearities as described in the previous paragraph.

In both cases, we consider the equations for the deviations Δs_{ij} and ΔV_i from the equilibrium values, and then write the equations as

$$D_t \Delta x = A(t), \tag{41}$$

On the left-hand side, x is either V_i or s_{ij} and D_t is a linear differential operator that contains all the linear terms of the equations. On the right hand-side, $A(t)$ is the sum of a linear forcing term Δy and all the nonlinear terms of the differential equation. $\Delta y(t)$ corresponds to Δs_{ij} for the step $\Delta V_i \leftarrow \Delta s_{ij}$, and to ΔV_j for $\Delta s_{ij} \leftarrow \Delta V_j$.

If there are no nonlinear terms, then we have an equilibrium Green's function g_0 that is obtained from

$$D_t g_0(t - t') = \delta(t - t'). \tag{42}$$

Given the Green's function g_0, we can write $\Delta x(t) = (g_0 * A)(t)$. We can write the latter expression for Δx also if $A(t)$ is a modified forcing term containing also nonlinear terms. In the nonlinear case, we should read off the nonequilibrium Green's function $g(t,t')$ from the expression of $\Delta x(t)$, using the definition

$$\Delta x(t) \equiv (g * \Delta y)(t) = \int dt_1\, g(t,t_1)\Delta y(t_1). \tag{43}$$

While this reading-off step is just a regrouping of terms, it yields the nonequilibrium Green's function, which is a useful formal device to compute other properties of the network, like the response functions.

The nonlinear differential equation for Δs_{ij} is (from Eq. 7 of the main text)

$$\frac{(\partial_t + a_r\phi_{\mathrm{eq}} + a_d)}{1 - s_{ij,\mathrm{eq}}}\Delta s_{ij}(t) = a_r\left(1 - \frac{\Delta s_{ij}(t)}{1 - s_{ij,\mathrm{eq}}}\right)\Delta\phi(V_j(t)). \tag{44}$$

By linearizing it around equilibrium we obtain the Green's function $\sigma_{0,ij}$ in Eq. (37), while using the above procedure and the definition $\Delta s_{ij}(t) \equiv \int dt_1 \sigma_{ij}(t,t_1)\Delta V_j(t_1)$, we obtain the expression for the nonlinear $\sigma_{ij}(t,t')$ (Eq. 8 in the main text).

The nonlinear equation for ΔV_i is (from Eq. 6 of the main text)

$$(\partial_t + \gamma_i + \sum_j \gamma^{\mathrm{g}}_{ij} + \sum_j \gamma^{\mathrm{s}}_{ij} s_{ij,\mathrm{eq}})\Delta V_i = \sum_j \gamma^{\mathrm{g}}_{ij}\Delta V_j - \sum_j \gamma^{\mathrm{s}}_{ij}\Delta s_{ij}(V_{i,\mathrm{eq}} - E_{ij} + \Delta V_i). \tag{45}$$

Again, its linearization leads to the equilibrium Green's functions in Eqs. (39) and (40). Focusing on one edge ij to obtain the nonequilibrium Green's function, we get

$$\begin{aligned}\Delta V_i(t) &= (g^{\mathrm{g}}_{0,ij} * \Delta V_j)(t) \\ &+ \int dt_1\, g^{\mathrm{s}}_{0,ij}(t-t_1)\left(1 - \frac{\Delta V_i(t_1)}{E_{ij} - V_{i,\mathrm{eq}}}\right)\Delta s_{ij}(t_1) \\ &= (g^{\mathrm{g}}_{0,ij} * \Delta V_j)(t) \\ &+ \int dt_1 dt_2\, g^{\mathrm{s}}_{0,ij}(t-t_1)\left(1 - \frac{\Delta V_i(t_1)}{E_{ij} - V_{i,\mathrm{eq}}}\right)(\sigma_{ij} * \Delta V_j)(t_1). \end{aligned} \quad (46)$$

From the above equation we can read off the nonequilibrium Green's function g_{ij} in Eq. 9 of the main text.

Finally, the nonequilibrium response function $\chi_{\alpha\beta}(t,t')$ in Eq. 10 of the main text is obtained applying Eqs. 3 and 4 (main text) to the nonequilibrium Green's function $\sigma_{\alpha\beta}(t,t')$:

$$\chi_{\alpha\beta}(t,t') = \sigma_{\alpha\beta}(t,t') + \int dq\, \frac{\delta\sigma_{\alpha\beta}(t,q)}{\delta V_\beta(t')}\Delta V_\beta(q). \quad (47)$$

Additional example and steps for calculations

We consider a more complex network with recurrence (Fig. 1a) and provide a detailed description of the steps involved in calculating that network's response functions. We consider the nonequilibrium state of the network induced by a 0.5 pA current pulse $I_\beta(t)$ injected into neuron β, which starts at $t = 0$ and lasts 1 s (the network parameters are described in the next section).

The response functions are calculated in two main steps. First, the nonequilibrium response function $f_{\alpha\beta}$ of the nonlinear edge $\alpha \Leftarrow \beta$ is calculated. This part of the calculation explicitly involves the nonlinearities, but it only involves the nonlinear edge $\alpha \Leftarrow \beta$, and the effective feedback edges $\beta \leftarrow \alpha$ and $\beta \leftarrow \beta$. Second, the nonequilibrium response function of any other effective edge is calculated via simple convolutions, without the need to re-run the nonlinear calculation.

In the first part we must compute $\overline{\chi}_{\alpha\beta}(t,t')$. For our *C. elegans* specific model, $\overline{\chi}_{\alpha\beta}(t,t')$ can be obtained using main text Eq. 3 and considering that $f_{\alpha\beta} = g^{\mathrm{s}}_{\alpha\beta} * \chi_{\alpha\beta}$, where $\chi_{\alpha\beta}$ is given by main text Eq. 10. To compute $\chi_{\alpha\beta}$, we need to know ΔV_β, which can be calculated by convolving the input current $I_\beta(t)$ with the nonequilibrium Green's function $G^{\mathrm{ext}}_\beta(t,t')$, which needs to be included in the explicitly nonlinear calculation using Eq. (24).

In the second part of the calculation, we use $\overline{\chi}_{\alpha\beta}(t,t')$ to compute any other response function of the network via Eq. (27) (Eq. 5 of the main text). This step does not involve any explicit knowledge of the form of the nonlinearities, because they are already captured by $\overline{\chi}_{\alpha\beta}(t,t')$, computed in the first step. As an example, we compute

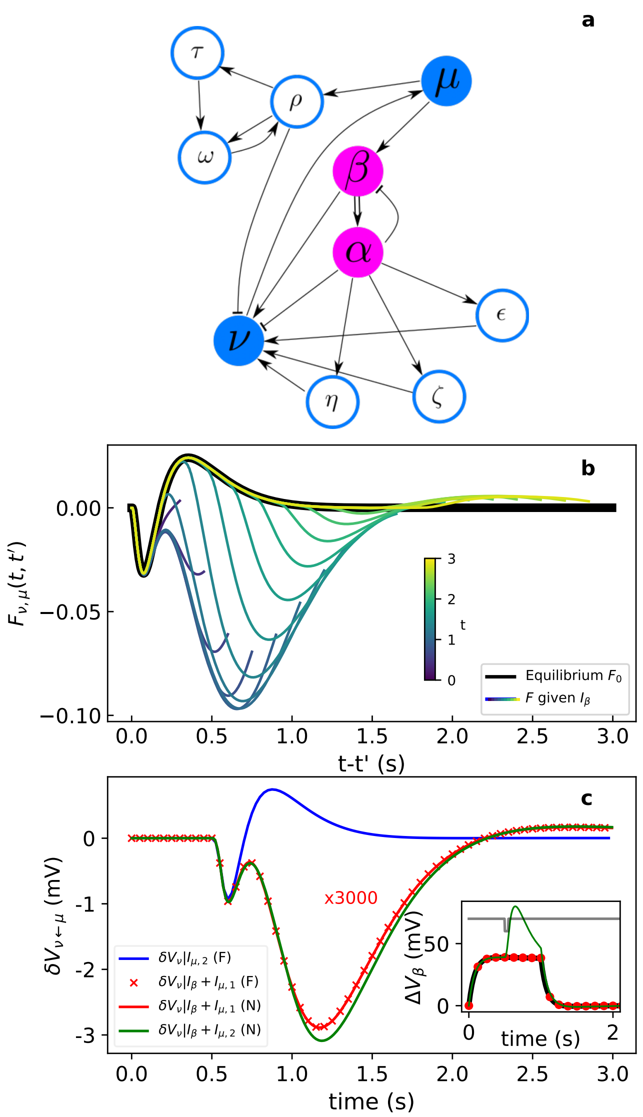

FIG. 1. **a** Scheme of the network. Neurons α and β (purple) are the only ones involved in the explicitly nonlinear calculation. The nonequilibrium response function $F_{\nu\mu}(t,t')$ is calculated for neurons μ and ν (blue). **b** Nonequilibrium and equilibrium $F_{(0,)\nu\mu}(t,t')$ for selected times t (colored and black curves, respectively). **c** $\delta V_{\nu\leftarrow\mu}$ induced by $I_{\mu,1}$ (red) and $I_{\mu,2}$ (green), calculated as $\Delta V_\nu\big|_{I_\beta+I_\mu} - \Delta V_\nu\big|_{I_\beta}$ (solid lines) and $F_{\nu\mu} * \delta V_\mu\big|_{I_\mu}$ (red crosses; Since $I_{\mu,2} = 3000 I_{\mu,1}$, the responses to the two currents calculated via $F_{\nu\mu}$ are simply proportional). $\delta V_{\nu\leftarrow\mu}$ calculated with the equilibrium response function $F_{0,\nu\mu}(t-t')$ (blue). **inset** ΔV_β produced by I_β only (black), by I_β plus $I_{\mu,1}$ (red dots, almost perfectly overlapping with the black line), and I_β plus $I_{\mu,2}$ (green curve). The gray curve is $I_{\mu,i}$ (scale not shown).

the nonequilibrium response function $F_{\nu\mu}(t,t')$, plotted in Fig. 1b for selected times (colored lines) together with the equilibrium $F_{0,\nu\mu}(t-t')$ (black line). To compute any response function of an edge (i,j) that does not end on β, we need to know $F_{\beta j}(t,t')$, the last factor of Eq. (27)

(Eq. 5 of the main text). To compute $F_{\beta j}(t,t')$, however, we do not need to know any other response function beyond $f_{\alpha\beta} = f_{0,\alpha\beta} + \overline{\chi}_{\alpha\beta}$ (because the left-hand side and the last factor of Eq. (27) are equal). Therefore, knowing $\overline{\chi}_{\alpha\beta}(t,t')$, we can compute any nonequilibrium $F_{\nu\mu}$ with just the additional step of computing $F_{\beta\mu}$.

With the response functions, we can then compute the response of the system to arbitrary additional stimuli, for example a current $I_\mu(t)$ injected into neuron μ. In general, this is possible only with additional stimuli that are sufficiently small, because it corresponds to a linearization of the system. However, as discussed above, because of the typical sigmoidal nonlinearities we find in neural networks, we can expect the nonequilibrium linearization to work over stimuli with a large range of amplitudes. We show this in Fig. 1c.

To check that the response function yields the correct result, we plot the responses of the system to a small, 0.05 s current pulse $I_{\mu,1}$, injected in neuron μ at time 0.5 s in addition to the current I_β. This current pulse into μ produces a very small additional change of the membrane potential V_β of the downstream neuron β, as can be seen in the inset of Fig. 1c. There the red dotted curve, computed including $I_{\mu,1}$, overlaps almost perfectly with the black curve, computed without $I_{\mu,1}$. Therefore, we expect the linear approximation to work well. We can check this by comparing the response $\delta V_{\nu\leftarrow\mu}$ of neuron ν to $I_{\mu,1}$ computed using the nonequilibrium response function (red crosses in Fig. 1c) with the one computed including $I_{\mu,1}$ in the explicitly nonlinear calculation (solid red line). As expected, they overlap very well.

The linear regime captured by the nonequilibrium response function holds also with large stimuli, in certain cases. To show this, we inject a much larger current $I_{\mu,2}$ into μ, which produces a downstream change in V_β that is of the same order as the one produced by the main current pulse I_β (green and black curves, respectively, in the inset of Fig. 1c). In such condition, it is not a given that the system can be linearized. Nevertheless, the response induced in ν by $I_{\mu,2}$ is still largely captured by the response function. In fact, $\delta V_{\nu\leftarrow\mu}$ calculated including $I_{\mu,2}$ in the explicit nonlinear calculation (Fig. 1c green curve), is very close to $\delta V_{\nu\leftarrow\mu}$ calculated via the response function (red curve, which, except for the appropriate rescaling by the ratio of $I_{\mu,2}$ and $I_{\mu,1}$, is the same as the response induced by $I_{\mu,1}$). As a comparison, the blue curve is the response $\delta V_{\nu\leftarrow\mu}$ calculated with the equilibrium response function, which produces a qualitatively wrong result.

Parameters of the numerical calculations

As parameters for the numerical calculations of the examples of the main text, we choose $C_i = 1$ pF, $E_{c,i} = -70$ mV, $\gamma_i = 10$ S/F for all the neurons and, for all the synapses, $\gamma^{\mathrm{s}}_{ij} = 10$ S/F, $E_{\mathrm{syn},ij} = 0$ mV, $a_{r,ij} = 5$, $a_{d,ij} = 5$, $\beta_{ij} = 125\,\mathrm{V}^{-1}$. $V_{\mathrm{th},ij} = V_{\mathrm{eq},j}$ for all the edges as in [12], except for edge (α,β), for which we set $V_{\mathrm{th},\alpha\beta} = -10$ mV. For the second example, the additional inhibitory edge $3 \vdash 1$ has $\gamma^{\mathrm{s}}_{31} = 20$ S/F, $E_{\mathrm{syn},31} = -70$ mV, and $a_{r,ij} = 1$, while all the other parameters of the synapse are equal to the ones above.

i,j	γ^s (S/F)	a_r	a_d	V_{th} (mV)	E_{syn} (mV)
β,μ	20	1	5	–	0
α,β	100	1	5	-10	0
ν,α	1000	1	2.5	–	-70
ϵ,α	6	1	2.5	–	0
ζ,α	6	1	2.5	–	0
η,α	6	1	2.5	–	0
ν,ϵ	16	1	5	–	0
ν,ζ	16	1	5	–	0
ν,η	16	1	5	–	0
ρ,μ	100	10	10	–	0
ω,ρ	100	10	10	–	0
τ,ρ	100	10	10	–	0
ρ,ω	10	10	10	–	0
ν,ρ	100	10	10	–	-70
β,α	10	1	5	–	-70
ν,β	10	3	5	–	0
μ,ν	1000	1	0.5	–	0

TABLE I. Parameters for the example reported in the Supplement (Fig. 1). Where V_{th} is unspecified, it means that it is set to $V_{\mathrm{eq},j}$.

For the example reported in the Supplement (Fig. 1), we have used $C_i = 1$ pF and $E_c = -70$ mV for all the neurons, and $\gamma_\mu = \gamma_\alpha = \gamma_\beta = \gamma_\nu = 10$, $\gamma_\epsilon = \gamma_\zeta = \gamma_\eta = 0.1$, and $\gamma_\rho = \gamma_\tau = \gamma_\omega = 20$. For all the synapses $\beta_{ij} = 125$, while the other synaptic parameters are in Table I. $V_{\mathrm{th},ij} = V_{\mathrm{eq},j}$ for all the edges as in [12] (unspecified $V_{\mathrm{th},ij}$ in Table I), except for $V_{\mathrm{th},\alpha\beta} = -10$ mV.

All the convolutions are performed using the Gregory integration scheme (of order 8), adapted from Ref. [13].

[1] Linz P. "Analytical and Numerical Methods for Volterra Equations". Studies in Applied and Numerical Mathematics, Society for Industrial and Applied Mathematics (1985).

[2] Brinkman B.A.W, Rieke F., Shea-Brown E., Buice M.A. "Predicting how and when hidden neurons skew measured synaptic interactions". PLoS Comput Biol 14(10): e1006490 (2018).

[3] Liu Q., Hollopeter G., Jorgensen E.M.: "Graded synaptic transmission at the *Caenorhabditis elegans* neuromuscular junction". PNAS 2009, 106(26):10823-10828

[4] Lindsay T.H., Thiele T.R., Lockery S.R.: "Optogenetic analysis of synaptic transmission in the central nervous

system of the nematode *Caenorhabditis elegans*". Nat. Comm. 2011, 2:306

[5] Narayan A., Laurent G., Sternberg P.W.: "Transfer characteristics of a thermosensory synapse in *Caenorhabditis elegans*". PNAS 2011, 108(23):9667–9672

[6] Liu M., Sharma A.K., Shaevitz J.W., Leifer A.M. "Temporal processing and context dependency in *Caenorhabditis elegans* response to mechanosensation" eLife 2018;7:e36419 doi.org/10.7554/eLife.36419

[7] Dobosiewicz M., Liu Q., Bargman C.I.: "Reliability of an interneuron response depends on an integrated sensory state". eLife 2019, 8:e50566 doi:10.7554/eLife.50566

[8] Ocker G.K., Josić K., Shea-Brown E., Buice M.A. "Linking structure and activity in nonlinear spiking networks". PLoS Comput Biol 13(6): e1005583 doi.org/10.1371/journal.pcbi.1005583

[9] Linderman S., Nichols A., Biel D., Zimmer M., Paninski L.: "Hierarchical recurrent state space models reveal discrete and continuous dynamics of neural activity in *C. elegans*". bioRXiv 2019, doi.org/10.1101/621540

[10] Costa A.C., Ahamed T., Stephens G.J.: "Adaptive, locally linear models of complex dynamics". PNAS 2019, 116(5):1501-1510

[11] Wicks S.R., Roehrig C.J., Ranking C.H.: "A Dynamic Network Simulation of the Nematode Tap Withdrawal Circuit: Predictions Concerning Synaptic Function Using Behavioral Criteria". The J. of Neurosci. 1996, 16(12):4017–4031

[12] Kunert J., Shlizerman E., Kutz J.N.: "Low-dimensional functionality of complex network dynamics: Neurosensory integration in the *Caenorhabditis elegans* connectome". Phys. Rev. E 2014, 89:052805

[13] Schüler M., Golež D., Murakami Y., Bittner N., Hermann A., Strand H.U.R., Werner P., Eckstein M. "NESSi: The Non-Equilibrium Systems Simulation package" arxiv.org:1911.01211
